# Clinical outcomes after post-operative radiotherapy for breast cancer patients presenting with ipsilateral supraclavicular metastasis: considerations on the cranial border of irradiation field

**DOI:** 10.1007/s12282-024-01644-9

**Published:** 2024-10-23

**Authors:** Xiaofang Wang, Xiaomeng Zhang, Li Zhang, Jin Meng, Wei Shi, Xingxing Chen, Zhaozhi Yang, Xin Mei, Xiaoli Yu, Zhen Zhang, Zhimin Shao, Xiaomao Guo, Jinli Ma

**Affiliations:** 1 Department of Radiation Oncology, Fudan University Shanghai Cancer Center, 270 Dong’an Road, Shanghai, 200032 China; 2 Department of Breast Surgery, Fudan University Shanghai Cancer Center, Shanghai, 200032 China; 3 Department of Oncology, Shanghai Medical College, Fudan University, Shanghai, 200032 China; 4 Shanghai Clinical Research Center for Radiation Oncology, Shanghai, China; 5 Shanghai Key Laboratory of Radiation Oncology, Shanghai, 200032 China

**Keywords:** Breast cancer, Radiotherapy, Supraclavicular metastasis, Cranial border

## Abstract

**Background:**

Disease recurrence at lower neck adjacent to ipsilateral supraclavicular (SCV) region represents a concern in locally advanced breast cancer patients presenting with SCV metastasis at diagnosis. This study aims to report the outcomes following post-operative radical radiation therapy and discuss the reasonable cranial border of the irradiation field for N3c patients.

**Methods:**

Between July 2016 and January 2022, a total of 268 patients were eligible for analysis. The endpoints included in-field and out-field cervical failures, local–regional recurrence-free survival (LRRFS), SCV recurrence-free survival (SRFS), distant metastasis-free survival (DMFS), relapse-free survival (RFS), and overall survival (OS).

**Results:**

During a median follow-up of 37 months (range 3–89 months), 17 patients (6.3%) developed local–regional recurrence as the first recurrence event, with 13 having concomitant distant-metastasis (DM); 56 patients (20.9%) had DM alone. The 3-year rates of LRRFS, SRF, DMFS, RFS, and OS were 92.3%, 94.5%, 74.5%, 73.0%, and 90.0%, respectively. 89.2% of patients received RT with the cranial border at the top of hyoid bone, and 95.1% of patients received a boost not exceeding the level of cricoid cartilage. A total of 11 patients (4.1%) developed ipsilateral SCV failure, and 3 patients (1.1%) experienced cervical failure, including 2 in-field failures and 1 out-field failure. Neoadjuvant systemic therapy (NST) was administered to 234 patients (87.3%). In the multivariate analysis, non-ypN0, triple-negative subtype and cT4 at diagnosis were predictors of worse SRFS and RFS in NST subgroup.

**Conclusion:**

Our findings suggest that radical RT with cranial border of irradiation field at the hyoid bone level lead to excellent local–regional control, and out-field cervical failure was rare. The irradiation field might not extend to mastoid process.

## Introduction

Ipsilateral supraclavicular lymph node (SCV LN) metastasis represents one of the manifestations of locally advanced breast cancer (LABC) and is classified as N3c in the 8th AJCC clinical staging system 1. In the last few decades, SCV metastasis has been considered potentially curable with multidisciplinary treatment, and with the state of art treatment, the survival was further improved in patients with LABC 2–6. Among the treatment options, radiotherapy (RT) plays a crucial role in the local control of the SCV and cervical region, which is typically not addressed through surgical intervention. Considering the high risk of recurrence in the supraclavicular fossa and lower neck adjacent to SCV, previous studies have suggested that the irradiated field could be extended cranially above the thyroid cartilage 7. However, the optimal superior border of RT for N3c patients remains controversial. Therefore, this study aims to report the survival outcomes and discuss the reasonable cranial border of the irradiation field for patients diagnosed with ipsilateral SCV metastasis but without distant metastasis under contemporary systemic treatment.

## Material and methods

### Patients

Among all the patients diagnosed with breast cancer and treated with RT at our institution between July 2016 and January 2022, a total of 366 patients were staged as N3c and underwent breast and axilla surgery. Of these, 98 patients were excluded for the following reasons: with positive lymph nodes exceeding cricoid cartilage level (n = 46), with double primary cancer (n = 10), lacking computed tomography (CT) or positron emission tomography/computed tomography (PET/CT) imaging at our institution (n = 27), with occult breast cancer (n = 5), without completing RT course as planned (n = 9), or with follow-up time less than 3 months (n = 1). The remaining 268 patients were included in the final cohort for analysis. The review of data was approved by the Ethics Committee and Institutional Review Board of our center.

Patient characteristics, including age, menopausal status, tumor laterality, location, details of surgery, histopathology, primary tumor size (T stage), number of axillary nodes removed and positive nodes, biological subtype, and Ki-67 index, were extracted from medical records. Hormonal receptor (HR) + was defined as ER + and/or PR + , and HR- as both ER- and PR-, as determined by immunohistochemistry (IHC) 8. A cutoff value of 1% was utilized to classify cases as positive or negative. HER2 was defined as positive if scored 3 + , negative if 1 + or 0, and indeterminate if 2 + on IHC. Indeterminate cases underwent further analysis using fluorescence in situ hybridization (FISH), with tumors considered HER2 positive if amplification was observed (ratio >  = 2.0) 9. Biological subtypes included HR + /HER2-, HR + /HER2 + , HR-/HER2 + , HR-/HER2- (triple negative BC, TNBC). All patients had imaging data, including CT, ultrasound, and/or PET/CT, which provided information on the status of the SCV LN and cervical LN at the time of initial diagnosis and post-treatment. Sites of positive regional LN, subregion of positive SCV LN, and residual SCV tumors after systemic therapy were reconfirmed by reviewing imagings mentioned above. For patients diagnosed with SCV metastasis via CT, CT scans at diagnosis and before RT were thoroughly reviewed by experienced physicians and radiologists, patients with SCV disease progressing on neoadjuvant chemotherapy or with suspicious positive SCV nodes that showed significant regression (> 50%) were included. The irradiation field, sites of boost, and RT dose were confirmed by reviewing the RT plan and record.

### Treatment

Typically, the treatment sequence applied was neoadjuvant systemic therapy (NST) followed by surgery. However, the sequence of surgery followed by adjuvant systemic therapy (AST) was allowed. All patients underwent irradiation to the ipsilateral chest wall (CW)/breast and regional nodes, including the supraclavicular, infraclavicular (ICV), internal mammary nodes (IMN) and undissected axilla LN (ALN), as well as cervical nodes. An additional boost was sequentially delivered to the SCV, other undissected positive LN, such as IMN, ICV and interpectoral (Rotter’s) LN, tumor bed for patients who received breast-conserving surgery (BCS), or mastectomy scars for patients with cT4 disease at the discretion of the treating physician. RTOG and ESTRO contouring guidelines were applied in our clinical practice, the posterior of the medial SCV was at anterior aspect scalene muscle; the posterolateral SCV was the fat space anterior to medial SCV, and posterior to scalenus or levator scapulae.

### Endpoints

Local-regional recurrence (LRR) was defined as the presence of clinical, radiographic, or pathological evidence of recurrence within ipsilateral CW/breast and/or regional nodes (i.e., ipsilateral ALN, SCV, ICV, or IMN). Distant metastasis (DM) was characterized as tumor relapse at sites other than locoregional. Ipsilateral cervical LN metastasis, a type of DM, indicated tumor relapse in the ipsilateral cervical LN beyond the level of the cricoid cartilage. The endpoints included in-field and out-field cervical failures, local-regional recurrence-free survival (LRRFS), SCV recurrence-free survival (SRFS), distant metastasis-free survival (DMFS), relapse-free survival (RFS), and overall survival (OS). LRRFS was measured from the date of the completion of RT to the time of LRR, the last visit or death. SRFS was defined as the interval from the completion of RT to the time of SCV recurrence, the last visit or death. DMFS was measured from the date of the completion of RT to the time of DM, the last follow-up or death. RFS was defined as the interval from the date of the completion of RT to the occurrence of LRR, DM, the last visit or death. OS was measured from the date of the completion of RT to the time of last follow-up or death.

### Statistical analysis

Patient characteristics were compared between subgroups using the Pearson’s χ2. The probabilities of LRRFS, RFS, DMFS, RFS, and OS were calculated using the Kaplan-Meier method. Both univariate and multivariate analyses were conducted using the Cox regression model to identify recurrence risk factors. The level of significance was set at p < 0.05 (two-sided), and all analyses were performed using SPSS 26.0.

## Results

### Patient and treatment characteristics

For the entire cohort, the median age was 48 years (range: 19–76 years). Most patients had invasive ductal carcinoma (n = 254, 94.8%). The clinical T stage was T1/T2 for 182 patients (67.9%), and T3/T4 for 86 patients (32.1%). All patients had pathologically confirmed axilla metastasis. 196 patients (73.1%) had involved SCV LN with cytological confirmation by fine needle aspiration, while the remaining patients were diagnosed by imaging, with 22 patients (8.2%) by PET/CT and 50 patients (18.7%) by CT. In addition to axilla and SCV involvement, 26 cases (9.7%) had IMN metastasis, 65 patients (24.3%) had ICV metastasis, and 13 patients (4.9%) had both. The majority of patients had SCV metastasis in the medial zone (n = 215, 80.2%), followed by both medial and posterolateral zone (n = 48, 17.9%). The distribution of sites of positive LN and subregion of positive SCV LN in the NST subgroup was similar to the entire cohort. HR was positive in 55.6% of patients (n = 149), and HER2 was positive in 43.7% of patients (n = 117) (Table 1).

**Table 1:** Patient characteristics at time of primary diagnosis and comparison between NST and AST subgroups

Parameters	Total (n = 268)	NST (n = 234)	AST (n = 34)	p
	N (%)	N (%)	N (%)	
Median age (range) (years)	48 (19–76)	48 (19–76)	53 (30–67)	/
Menopausal status				
Pre/peri-	171 (63.8)	157 (67.1)	14 (41.2)	0.003
Post-	97 (36.2)	77 (32.9)	20 (58.8)	
Tumor laterality				
Left	162 (60.4)	139 (59.4)	23 (67.6)	.358
Right	106 (39.6)	95 (40.6)	11 (32.4)	
Tumor location				
Medial or central	74 (27.6)	66 (28.3)	8 (23.5)	< 0.001
Outer	146 (54.5)	134 (57.3)	12 (35.3)	
Above/below the nipple	27 (10.1)	24 (10.3)	3 (8.8)	
Multi-quadrant	12 (4.5)	9 (3.8)	3 (8.8)	
Unknown	9 (3.4)	1 (0.4)	8 (23.5)	
Tumor histopathology				
IDC	254 (94.8)	223 (95.3)	31 (91.2)	0.307
ILC	8 (3.0)	7 (3.0)	1 (2.9)	
Special subtypes	6 (2.2)	4 (1.7)	2 (5.9)	
T stage				
cT1	36 (13.4)	27 (11.5)	9 (26.5)	0.006
cT2	146 (54.5)	127 (54.3)	19 (55.9)	
cT3	37 (13.8)	31 (13.2)	6 (17.6)	
cT4	49 (18.3)	49 (20.9)	0 (0.0)	
cT4d	19 (7.1)	19 (8.1)	0 (0.0)	/
Sites of regional nodes				
SCV + ALN alone	164 (61.2)	146 (62.4)	18 (52.9)	0.540
SCV + ALN + IMN	26 (9.7)	21 (9.0)	5 (14.7)	
SCV + ALN + ICV	65 (24.3)	55 (23.5)	10 (29.4)	
SCV + ALN + IMN + ICV	13 (4.9)	12 (5.1)	1 (2.9)	
Biological subtype				
HR + /HER2-	104 (38.8)	86 (36.8)	18 (52.9)	0.262
HR + /HER2 +	45 (16.8)	39 (16.7)	6 (17.6)	
HR-/HER2 +	72 (26.9)	66 (28.2)	6 (17.6)	
HR-/HER2-	47 (17.5)	43 (18.4)	4 (11.8)	
Ki-67 value				
< 30%	91 (34.0)	78 (33.3)	13 (38.2)	0.573
≥ 30%	177 (66.0)	156 (66.7)	21 (61.8)	
Diagnose of positive SCV LN				
Confirmed by pathology	196 (73.1)	184 (78.6)	12 (35.3)	< 0.001
PET/CT suspicion	22 (8.2)	13 (5.6)	9 (26.5)	
Imaging suspicion	50 (18.7)	37 (15.8)	13 (38.2)	
Subregion of positive SCV LN				
Medial	215 (80.2)	182 (77.8)	33 (97.1)	0.014
Posterolateral	5 (1.9)	4 (1.7)	1 (2.9)	
Medial + posterolateral	48 (17.9)	48 (20.5)	0 (0.0)	

*NST* neoadjuvant systemic therapy, *AST* adjuvant systemic therapy, *IDC* invasive ductal carcinoma, *ILC* invasive lobular carcinoma, *ALN* axillary lymph nodes (level I-II), *SCV* supraclavicular, *ICV* infraclavicular, *IMN* internal mammary nodes

A total of 234 patients (87.3%) received NST. Of these, 86 patients (36.8%) were HR + /HER2-, 39 patients (16.7%) were HR + /HER2 + , 66 patients (28.2%) were HR-/HER2 + , and 43 patients (18.4%) were HR-/HER2-. All HER2-positive patients received anti-HER2 therapy. Following NST, 89 patients (38.0%) achieved pathological complete response (pCR) of primary tumor in the breast (ypT0); 106 patients (45.3%) achieved nodal pCR in the axilla (ypN0); and 73 patients (31.2%) achieved pCR in both the breast and axilla tumors (ypCR) (Table 2).

**Table 2:** Treatment and pathological response in NST subgroup

Parameters	N = 234
	No. (%)
Neoadjuvant systemic treatment	
Median no. of cycles of neo-chemotherapy (range)	6 (1–16)
Endocrine therapy alone	1 (0.4)
Anti-HER2 therapy	107 (45.7)
ypT	
ypT0	89 (38.0)
ypT1	96 (41.0)
ypT2	41 (17.5)
ypT3	7 (3.0)
ypT4	1 (0.4)
ypN	
ypN0	106 (45.3)
ypN1	41 (17.5)
ypN2	39 (16.7)
ypN3	48 (20.5)
ypCR	
Yes	73 (31.2)
No	161 (68.8)

*NST* neoadjuvant systemic therapy, *ypCR* pathological complete response

For the entire cohort, 91.8% of patients underwent modified radical mastectomy (MRM), and none of them received supraclavicular dissection. Adjuvant chemotherapy was administered to 139 patients (51.9%), endocrine therapy to 149 patients (55.6%), and anti-HER2 therapy to all HER2 positive patients at diagnosis and two patients who showed HER2 positive post-NST (n = 119, 44.4%) (Table 3).

**Table 3:** Treatment summary of surgery, AST and RT

Parameters	Total (n = 268)
	No. (%)
Surgery	
MRM	246 (91.8)
M + SLNB	3 (1.1)
BCS + ALND	19 (7.1)
AST	
Chemotherapy	139 (51.9)
Anti-HER2 therapy	119 (44.4)
Endocrine therapy	149 (55.6)
Radiation treatment	
CTV of SCV	
Medial	7 (2.6)
Posterolateral	0 (0)
Medial + posterolateral	261 (97.4)
Upper level of SCV CTV	
Cricoid cartilage	12 (4.5)
Hyoid bone	239 (89.2)
Mastoid process	17 (6.3)
Sites of SCV boost	
None	4 (1.5)
Medial	211 (78.7)
Posterolateral	4 (1.5)
Medial + posterolateral	49 (18.3)
Upper level of SCV boost	
Cricoid cartilage	255 (95.1)
Hyoid bone	9 (3.4)
Boost of LN except SCV	
None	4 (1.5)
ALN	2 (0.7)
IMN ± ALN	22 (8.2)
ICV ± ALN	56 (20.9)
IMN + ICV ± ALN	10 (3.7)
Boost of TB or CW	48 (17.9)
Dose of PTV (Gy/Fx)	50/25
Median boost dose (Gy/Fx) (range)	10/5 (6/3–16/8)
≥ 16 Gy	4 (1.5)
≥ 10 Gy, < 16 Gy	257 (95.9)
< 10 Gy	7 (2.6)

*AST* adjuvant systemic therapy, *MRM* modified radical mastectomy, *M* simple mastectomy, *BCS* breast-conserving surgery, *SLNB* sentinel node biopsy, *ALND* axillary lymph node dissection, *CTV* clinical target volume, *SCV* supraclavicular, *ALN* axillary lymph nodes (level I-II), *ICV* infraclavicular, *IMN* internal mammary nodes, *TB* tumor bed, *CW* chest wall, *PTV* planning target volume

### Radiotherapy

Pre-RT evaluation showed that 72 (26.9%) patients had residual nodal disease of ≥ 0.5 cm on CT, or showed nodal hypermetabolic activity on PET/CT. Physicians contoured the medial and posterolateral zones of SCV as clinical target volume (CTV) in the majority of the patients (n = 261, 97.4%). The cranial border of SCV CTV was basically at the level of cricoid cartilage. 95.5% of people (n = 256) extended to a higher cranial border than the cricoid cartilage. The cranial border of cervical CTV was extended to the level of hyoid bone in 89.2% of patients (n = 239), and to the level of mastoid process in 6.3% of patients (n = 17). Among patients who received irradiation with the cranial border at the level of the cricoid cartilage, hyoid bone, and mastoid process, there was no statistically significant difference in the distribution of patient characteristics, including age (p = 0.492), cT stage (p = 0.505), site of nodes involved (p = 0.758), biological subtypes (p = 0.957), subregion of positive SCV LN (p = 0.633) at diagnosis, and pCR of the breast (p = 0.728) and axillary nodes (p = 0.574) after NST and surgery. 264 patients (98.5%) received a boost to SCV, including medial SCV in 211 patients (78.7%), medial and posterolateral SCV in 49 patients (18.3%), and posterolateral SCV in 4 patients (1.5%). Among the 4 patients who did not receive a boost to SCV, 2 of them refused to continue after completing 25 fractions of loco-regional RT due to grade 3 radiation dermatitis; the other 2 patients underwent PET/CT scans before RT, which revealed no residual tumors in the SCV, so individualized treatment without a boost to SCV was applied. The dose to the planning target volume (PTV) for chest wall/breast and regional nodes was 50 Gy/25Fx, the median boost dose was 10 Gy/5Fx (range, 6 Gy/3Fx-16 Gy/8Fx), all delivered with intensity-modulated RT (IMRT) technique (Table 3).

### Survival

**Fig. 1:**
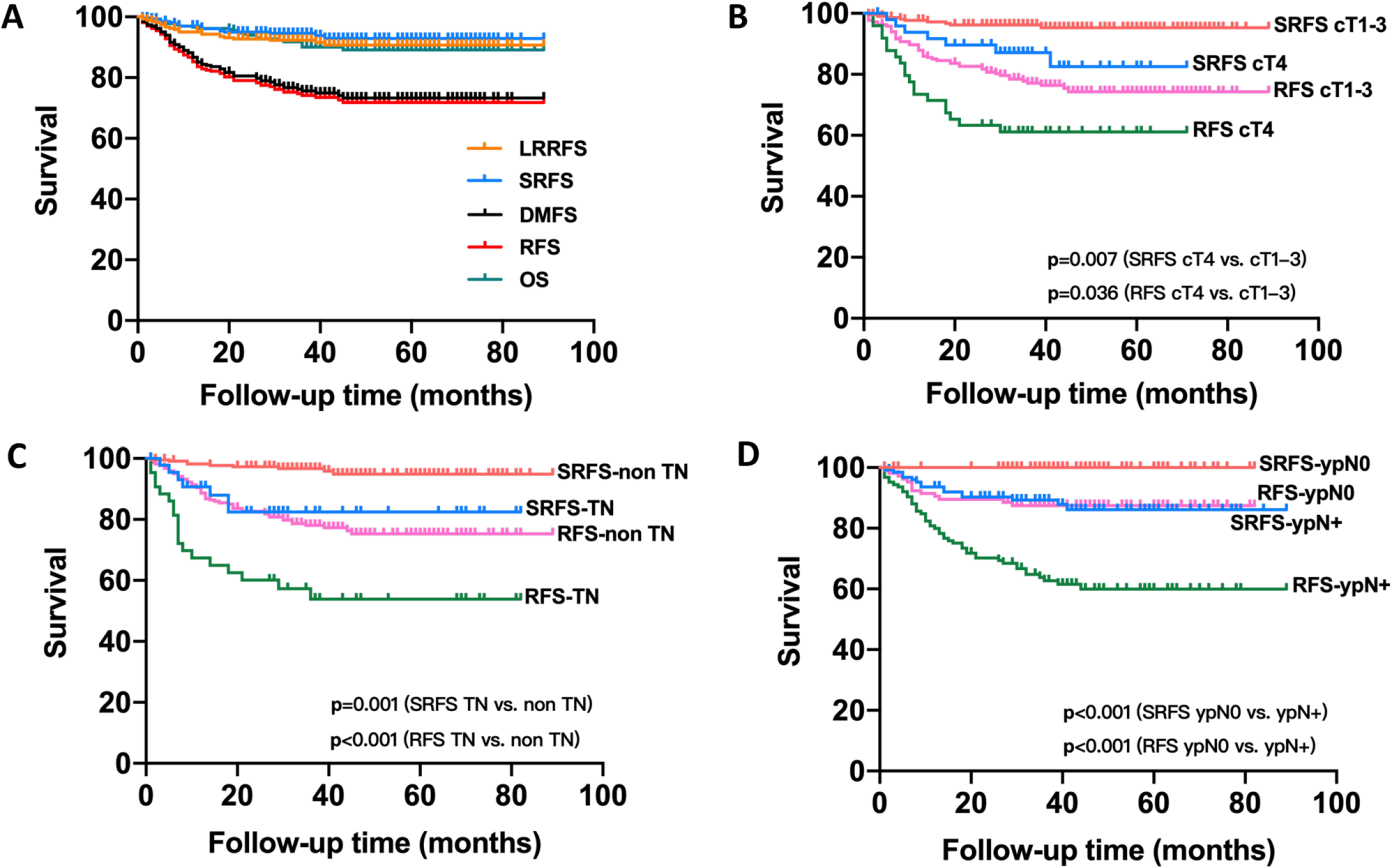
**A** LRRFS, SRFS, DMFS, RFS and OS curves for the entire cohort. **B** SRFS and RFS for patients with cT1-3 and cT4 diseases at diagnosis for the entire cohort. **C** SRFS and RFS for patiens with TN and non-TN breast cancer for the entire cohort. **D** SRFS and RFS for patients with ypN0 and ypN+ in NST group.

With a median follow-up of 37 months (range, 3–89 months), 73 patients experienced any recurrence. Of these, 17 patients developed LRR as the first recurrence event, with 13 having concomitant DM; 56 patients had DM alone (Table 4). 25 patients had died due to breast cancer. For the entire cohort, the 3-year rates of LRRFS, SRF, DMFS, RFS, and OS were 92.3%, 94.5%, 74.5%, 73.0%, and 90.0%, respectively; and the 5-year rates of LRRFS, SRF, DMFS, RFS, and OS were 90.7%, 92.9%, 72.2%, 70.7%, and 89.1%, respectively (Fig. 1A).

**Table 4:** Patterns of progression

Progression at any site	Parameters	Total (%) (n = 73)
	LRR alone	4 (5.5)
	LRR + DM	13 (17.8)
	DM alone	56 (76.7)
Locoregional recurrence	Parameters	Total (%) (n = 17)
	SCV	11 (64.7)
	SCV alone	7
	SCV + ALN	2
	SCV + CW	2
	Axilla	1 (5.9)
	CW or Breast	5 (29.4)

*LRR* local–regional recurrence, *DM* distant metastasis, *SCV* supraclavicular, *CW* chest wall, *ALN* axillary lymph nodes (level I-II)

For AST and NST subgroups, the 3-year rates of LRRFS, SRF, DMFS, RFS, and OS were 90.7% vs. 92.5% (p = 0.856), 96.9% vs. 94.2% (p = 0.444), 76.5% vs. 74.3% (p = 0.949), 73.5% vs. 73.0% (p = 0.777), 89.8% vs. 90.0% (p = 0.935), respectively.

For patients diagnosed SCV positive via pathology and imaging, the 3-year LRRFS, SRF, DMFS, RFS, and OS were 93.6% vs. 88.7% (p = 0.276), 95.1% vs. 92.8% (p = 0.650), 75.4% vs. 71.9% (p = 0.443), 74.4% vs. 69.1% (p = 0.286), and 90.2% vs. 89.6% (p = 0.549), respectively.

For HR + /HER2-, HR + /HER2 + , HR-/HER2 + , HR-/HER2- patients, the 3-year LRRFS was 95.1%, 97.6%, 98.6%, and 72.1% (p < 0.001), respectively; the 3-year SRF was 94.0%, 100%, 98.6%, and 82.4% (p = 0.001), respectively; and the 3-year DMFS was 72.9%, 90.7%, 79.1%, and 55.7% (p < 0.001), respectively. Correspondingly, the 3-year RFS was 71.9%, 90.7%, 79.1%, and 49.3% (p < 0.001), respectively. The 3-year OS was 88.8%, 95.5%, 96.3%, and 77.7% (p = 0.002), respectively.

### SCV and cervical recurrence

94.1% of LRR and 95.7% of DM events occurred in the first 3 years from RT. 17 patients experienced LRR as the first recurrence event, including 11 patients (64.7%) who developed SCV recurrence. Among these with SCV recurrence, 3 (27.3%) patients had isolated LRR, and 8 (72.7%) patients had concomitant DM; pre-RT CT scans revealed that 6 patients had residual SCV diseases of less than 0.5 cm, while the others had residual SCV diseases of 0.5 cm or more, or showed hypermetabolic activity on PET/CT. All of the 11 patients suffered SCV failure in the irradiation field, which was delivered a dose of 50 Gy in 25 fractions. Among those, 9 patients relapsed in the SCV boost area with a total RT dose of 60 Gy/30Fx, 1 patient out of the boost area, and another 1 patient did not receive a SCV boost.

3 patients experienced ipsilateral cervical LN failure, including 2 within the irradiation field and 1 outside the irradiation field. All of these patients exhibited concomitant DM at sites other than the cervical LN. Furthermore, two out of these three patients experienced a concomitant recurrence in the SCV.

For patients with the cranial border at the level of hyoid bone, the 3-year LRRFS, SRF, DMFS, RFS, and OS were 91.7%, 94.2%, 73.4%, 71.8%, and 89.2%, respectively; and the 5-year LRRFS, SRF, DMFS, RFS, and OS were 89.9%, 92.4%, 71.7%, 70.1%, and 88.1%, respectively. No statistically significant differences were observed in terms of LRRFS (p = 0.513), SRF (p = 0.662), DMFS (p = 0.379), RFS (p = 0.337), and OS (p = 0.369) among patients with the cranial border at levels of hyoid bone, mastoid process, and cricoid cartilage.

### Risk factors

The correlation of SRFS and RFS with various prognostic factors for the entire cohort is shown in Table 5. In univariate analysis, TNBC (p = 0.001) (Fig. 1C) and staged at cT4 at diagnosis (p = 0.007) (Fig. 1B) were correlated with a worse SRFS. TNBC (p < 0.001) (Fig. 1C), cT4 stage at diagnosis (p = 0.036) (Fig. 1B) and right breast cancer (p = 0.019) were associated with a shorter RFS. The multivariate analysis revealed that TNBC (HR, 1.836, 95% CI 1.309–2.576, p < 0.001 for SRFS; HR:1.467, 95% CI 1.241–1.734, p < 0.001 for RFS) and cT4 (HR, 1.677, 95% CI 1.196–2.349, p = 0.003 for SRFS; HR:1.211, 95% CI 1.012–1.450, p = 0.037 for RFS) were predictors of shorter SRFS and RFS.

**Table 5:** Univariate and multivariate analyses of patient clinical and treatment-related factors for SRFS and RFS for the entire cohort

Parameters	3-year SRFS (%) (Entire cohort)	*P*		3-year RFS (%) (Entire cohort)	*P*	
		UVA	MVA		UVA	MVA
Age		0.242	/		0.183	/
≤ 40	92.1			67.0		
> 40	95.3			75.0		
Tumor laterality		0.054	0.161		0.019	0.055
Left	96.8			78.4		
Right	91.0			64.8		
Tumor location		0.764	/		0.684	/
Medial or central	95.8			73.7		
Outer	93.5			71.1		
Above/below the nipple	96.2			83.3		
Multi-quadrant	91.7			66.7		
cT stage		0.007	0.003		0.036	0.037
cT1-3	96.2			75.7		
cT4	87.1			61.1		
Sites of regional nodes		0.569	0.464		0.341	0.206
SCV + ALN alone	95.5			75.6		
SCV + ALN + IMN/ICV	93.0			68.8		
Subregion of positive SCV LN		0.630	0.954		0.694	0.894
Medialis	95.1			73.7		
Posterolateral	100.0			80.0		
Medialis + Posterolateral	91.1			68.7		
Biological subtype		0.001	< 0.001		< 0.001	< 0.001
HR-/HER2-	82.4			49.3		
Other subtypes	96.7			78.0		
Ki67 value		0.058	0.320		0.080	0.471
< 30%	98.9			80.1		
≥ 30%	92.2			69.4		
Upper level of SCV CTV		0.662	0.607		0.337	0.235
Cricoid cartilage	100.0			75.0		
Hyoid bone	94.2			71.8		
Mastoid process	94.1			88.2		
Upper level of SCV boost		0.302	0.853		0.743	0.920
Cricoid cartilage	95.0			73.2		
Hyoid bone	88.9			66.7		

*RFS* relapse-free survival, *SRFS* SCV recurrence-free survival, *UVA* univariate analyses, *MVA* multivariate analysis, *ALN* axillary lymph nodes (level I-II), *ICV* infraclavicular, *IMN* internal mammary nodes, *CTV* clinical target volume

The correlation of SRFS and RFS with various prognostic factors for the NST subgroup was analyzed. In univariate analysis, TNBC (p = 0.001), cT4 at diagnosis (p = 0.012), non-ypT0 (p = 0.039), non-ypN0 (p < 0.001) (Fig. 1D), and right breast cancer (p = 0.038) were associated with worse SRFS; TNBC (p < 0.001), cT4 at diagnosis (p = 0.030), non-ypT0 (p = 0.001), and non-ypN0 (p < 0.001) (Fig. 1D) were associated with poorer RFS. None of the patients who achieved ypN0 had either SCV or ipsilateral cervical failure. Besides non-ypN0, TNBC (HR, 2.423, 95% CI 1.513–3.882, p < 0.001) and cT4 at diagnosis (HR, 1.674, 95% CI 1.135–2.470, p = 0.009) were also associated with short SRFS time on multivariate analysis. Furthermore, non-ypN0 (HR, 4.031, 95% CI 2.179–7.457, p < 0.001) (Fig. 1^1^D), TNBC subtype (HR, 1.556, 95% CI 1.303–1.858, p < 0.001), and cT4 stage (HR, 1.255, 95% CI 1.048–1.504, p = 0.014) were independent predictors of a worse RFS interval.

## Discussion

Previous studies indicated that supraclavicular surgery plus RT did not improve the prognosis of breast cancer patients with synchronous ipsilateral supraclavicular metastasis in comparison with RT alone 10–12. Radical RT for the SCV fossa remains a viable option to achieve optimal regional control. It is essential to define the volume and dose for SCV irradiation, balancing tumor control and complications. The National Comprehensive Cancer Network (NCCN) recommends that a supplemental boost of RT can be delivered to grossly involved or enlarged lymph nodes that have not been surgically addressed 13. A boost of 10-16 Gy to SCV nodes was routinely prescribed in clinical practice 3, 4, 14, 15. Our previous study reported that an SCV boost to 60 Gy did not increase the incidence of brachial plexus (BP) neuropathy-related symptoms, indicating that the toxicity to the BP was acceptable 16. Mapping studies from Brown et al 17, Jing et al 18 and Li et al 19 indicated that the SCV CTV might be more extensive than RTOG 20 and ESTRO 21 volume for patients with ipsilateral SCV metastasis. The TransAtlantic Radiation Oncology Network (TRONE) recommended applying the RADCOMP atlas 22 for contouring the CTV of the SCV fossa, which includes the medial and posterolateral zones of the supraclavicular fossa 7. The European Society for Radiotherapy and Oncology (ESTRO) describes the target contouring for N3 patients as “considering margin expansions of 10–20 mm around the pathologically involved nodes” 7. However, there has been limited research in the setting of the cranial border of the radiation field. In this study, patients were treated according to the consensus, with 97.4% of patients receiving SCV irradiation to ≥ 60 Gy, and 97.4% of patients receiving radiation to medial and posterolateral SCV regions. Regarding systemic therapy, the vast majority of patients received standard chemotherapy, anti-HER2 therapy, and endocrine therapy, or participated in clinical trials. Under this circumstance, we reported the survival of patients with SCV metastasis and explored the optimal cranial border of the radiation field.

In this study, a total of 73 patients experienced any recurrence, including 56 patients with DM alone, 4 patients with LRR alone, and 13 patients with simultaneous LRR and DM as the first recurrence. The 3-year rates of LRRFS, SRF, DMFS, RFS, and OS for were 92.3%, 94.5%, 74.5%, 73.0%, and 90.0%, respectively; the corresponding 5-year rates were 90.7%, 92.9%, 72.2%, 70.7%, and 89.1%, respectively; which were comparable with previous studies. For instance, Hae Jin Park et al. analyzed 55 patients diagnosed with cN3c disease via PET/CT. With a median follow-up of 38 months, the 5-year LRRFS, DFS, and OS were 80%, 60%, and 79%, respectively 14. Huang EH et al. reviewed the data from 71 N3c patients, with a median follow of 3.7 years, the 5-year SRF, LRRFS, DFS, and OS were 90%, 77%, 30%, and 47%, respectively 3. Kevin Diao et al. analyzed the data from 173 patients, with a median follow-up time of 2.8 years, the 3-year rates of OS, SCRFS, LRRFS, DMFS, and RFS were 79%, 95%, 89%, 64%, and 61%; the corresponding 5-year rates were 73%, 95%, 86%, 56%, and 50%, respectively 15.

For this cohort of patients, the cranial border of irradiation field was most commonly set at the hyoid bone (n = 239, 89.2%), followed by the mastoid process (n = 17, 6.3%), and the cricoid cartilage (n = 12, 4.5%). Although the survival was observed not to alter with cranial borders, the sample sizes in the cricoid cartilage and mastoid process subgroups might be too limited to detect true survival differences. Few studies described the cranial border of irradiated field and reported the in-field and out-field cervical failures for N3c patients who received definitive RT for SCV area. Diao et al. 15 reported delivering a boost to the SCV and cervical zone with the superior border at the bottom of the mastoid process. In our study, 89.2% of patients received RT with the cranial border at the top of the hyoid bone, and 95.1% of patients received a boost not exceeding the level of cricoid cartilage. The ipsilateral cervical irradiated field was smaller in our study than in Diao’s. However, tumor control of SCV and RFS were comparable. Advanced, in this study, 1.3% of patients (n = 3) experienced ipsilateral cervical LN metastasis after RT among the 239 patients who had the cranial border at the hyoid bone level. Among which, 2 patients suffered with in-field failure and 1 patient with out-field failure. Concomitantly, all of the patients exhibited DM at other sites. The cervical LN recurrence pattern revealed that with the cranial border at the level of hyoid bone, cervical failure was rare and the cervical irradiation might be sufficient. However, for patients with aggressive diseases who developed cervical LN and SCV metastasis, higher prescription dose may lead to better regional control. On the other hand, as cervical LN metastasis is a condition of DM, better use of systemic therapy might be more important to improve survival.

NST continues to play a role in the initial management of LABC, and potential benefits include tumor downstaging to facilitate breast and axilla surgery, improved prognosis, and de-escalation of nodal radiation for patients who experience nodal pCR 23, 24. In this study, 87.3% of N3c patients received NST. Survival analyses showed that there was no statistically significant difference in terms of LRRFS, SRF, DMFS, RFS, and OS when compared between NST and AST subgroups. Patients staged at cT4 at diagnosis and with TNBC had worse RFS and SRFS for the entire cohort, consistent with previous studies 4, 15. For these patients, intensifying systemic therapy and regional RT may be a strategy to improve survival. In the NST subgroup, 45.3% of patients achieved axillary nodal pCR, among whom none progressed with ipsilateral SCV or cervical LN recurrence. ypN0 was also found to be a predictor of better RFS, with the 3-year RFS of 87.5% vs. 61.3%, and 5-year RFS of 87.5% vs. 58.6% for ypN0 and non-ypN0 patients, respectively. Given the favorable clinical outcomes of ypN0 patients, de-escalation of RT, e.g., the omission of lower neck irradiation, and/or the omission of boost to SCV with clinical CR of gross disease, might be considered in future prospective trials.

This study represents one of the largest series to date of initial SCV metastasis cases treated with radical RT after breast and axilla surgery, and to discuss the reasonable cranial border of SCV irradiation. However, our study still has certain limitations, including its retrospective nature, limited follow-up time, and imbalanced samples among three subgroups with different cranial borders of SCV field. Comparing our finding with historical literature, the survival data revealed that our smaller irradiated field, with the cranial border at the level of hyoid bone, did not compromise SCV and distant tumor control. Further, irradiation field with lower cranial border might potentially lead to less adverse events, such as mucositis, dysphagia, esophagitis, xerostomia, and neck fibrosis, etc.

## Conclusion

Following breast and axilla surgery, radical RT with cranial border of irradiation field at the hyoid bone level might be sufficient under contemporary systemic treatment. The irradiation field might not extend to mastoid process. For patients with ypN0 and non-TNBC subtype, de-escalation of RT could be considered to optimize therapeutic index in future prospective trials.

## Data Availability

Research data are stored in an institutional repository and will be shared upon request to the corresponding author.
